# Charcot foot reconstruction with combined internal and external fixation: case report

**DOI:** 10.1186/1749-799X-5-7

**Published:** 2010-02-11

**Authors:** Claire M Capobianco, Crystal L Ramanujam, Thomas Zgonis

**Affiliations:** 1 Division of Podiatric Medicine and Surgery, Department of Orthopaedic Surgery, University of Texas Health Science Center at San Antonio, 7703 Floyd Curl Drive, San Antonio, TX, 78229, USA

## Abstract

Charcot neuroarthropathy is a destructive and often-limb threatening process that can affect patients with peripheral neuropathy of any etiology. Early recognition and appropriate management is crucial to prevention of catastrophic outcomes. Delayed diagnosis and subsequent pedal collapse often preclude successful conservative management of these deformities and necessitate surgical intervention for limb salvage. We review the current literature on surgical reconstruction of Charcot neuroarthropathy and present a case report of foot reconstruction with combined internal and external fixation methods.

## Background

Charcot neuroarthropathy (CN) was originally described in 1868 [1] as a rare affliction of patients with leprosy and alcoholism that resulted in fragmentation, collapse, and subsequent deformity of the pedal joints in the neuropathic lower extremity. The demographics of patients with CN today reflect the exponential rise in the prevalence of diabetes mellitus over the last twenty years. Charcot neuroarthropathy develops in approximately 0.3-7.5% of patients with diabetic peripheral neuropathy, and has significant long term prognostic implications [2,3]. Charcot collapse of pedal architecture predictably progresses to plantar deformity, ulceration, and ultimately, if not addressed, infection and amputation. Ten to fifteen percent of patients with diabetes mellitus will undergo lower extremity amputation in their lifetime [4], with CN deformity a clear amputation risk factor.

Although the pathophysiology of the disease remains unknown, two principal theories have been proposed. The neurotraumatic theory postulates that repetitive microtrauma in the insensate foot results in unrecognized subchondral fractures that, with continued activity, lead to subsequent joint fragmentation and subluxation. On the other hand, the neurovascular theory focuses on the autonomic dysfunction associated with peripheral neuropathy. Pathologically increased sympathetic activity results in hyperemia, which potentiates bone resorption and subsequent periarticular fractures and joint subluxation [5,6]. An imbalance in osteoclastic and osteoblastic activity is thought to contribute to the pathogenesis of the process [7], and research continues in this area.

In the acute CN stage, patients present with a unilateral erythematous and edematous lower extremity, which may or may not be painful. Patients often cannot recall a specific traumatic event, but a careful history may reveal an episode of seemingly benign increased activity prior to the onset of symptoms. Deformity may or may not be present in the foot and, in the truly acute stage, radiographic abnormalities may be absent. Clinically, elevation of the affected limb results in diminished appearance of erythema, unless a coexistent ulcer and infectious process is also present. Strict and complete non-weight bearing and cast immobilization of the affected limb is crucial to management of the acute CN foot.

In the coalescent CN stage, 3-6 months after initial appearance, patients typically present with rocker bottom foot deformity, often with plantar ulceration at bony prominences. The ulcerations are usually chronic in nature and have been refractory to previous wound care. Radiographs taken in the subacute or coalescent stage often demonstrate subchondral cyst formation, peri-articular fragmentation and severe dislocation and subluxation of the midfoot and/or rearfoot and ankle joints. Charcot neuroarthropathy most commonly affects the tarsometatarsal joints (27-60%), but may also affect the Chopart joint complex (30%), the subtalar (35%) and/or ankle (9%) joints and, rarely, the calcaneal tuberosity [8]. The prognosis of rearfoot and ankle CN deformity is universally accepted as poorer than that of forefoot and midfoot deformities.

Controversy exists in the literature regarding surgical intervention on CN foot and ankle deformities. Most authors advocate intervention in the coalescent or consolidative CN stages [9,10], but early arthrodesis and open reduction and internal/external fixation during the developmental stage have been reported [9,11,12]. The authors recognize the highly individualized nature of each patient with CN deformity and hence do not advocate a generalized treatment algorithm for the Charcot foot. Surgical intervention is recommended when the patient's deformity is recalcitrant to appropriate conservative treatment and potentiates an ulceration, is not amenable to bracing or custom shoe gear, when osteomyelitis is present, or when the deformity endangers the intact skin envelope. Published literature has reported greater than 90% limb salvage rates after major foot and ankle reconstruction in patients with CN deformity [10,13], but the importance of proper patient selection, exacting technique and familiarity with the natural history of the disease cannot be underestimated.

## Case Report

A 48-year-old male presented to clinic with chief concern of a painful right foot. The patient related a history of foot injury sustained during exercise on a treadmill approximately one year previously. He had been treated by an outside practitioner with four months of cast immobilization, but experienced continued pain, edema, and instability from midfoot collapse. The patient's medical history was significant for type 2 diabetes mellitus, peripheral neuropathy, hypertension, morbid obesity, and gastritis. He had a history of surgery to his back, left knee, and left shoulder under general anesthesia without complications. His family history was significant for diabetes mellitus and coronary artery disease. On presentation, a review of systems was significant only for his chief complaint.

At initial evaluation, the patient's vital signs were stable and he was afebrile. His cardiopulmonary exam revealed no abnormalities. The focused lower extremity exam was significant for midfoot edema, rocker bottom deformity, notable plantar prominences along the tarsometatarsal joints with corresponding preulcerative lesions, and severe forefoot abduction. There were no open wounds or signs of acute infection. Manual muscle strength testing of all extrinsic muscles of the foot and ankle revealed no deficits. Dorsalis pedis and posterior tibial pulses were strongly palpable, capillary refill time was immediate to all digits and pedal hair was present. Sensation to light touch was diminished to all nerve distributions of the foot bilaterally to the ankle level. Vibratory sensation was markedly diminished to the first metatarsophalangeal joint bilaterally and the patient demonstrated profound loss of protected sensation via Semmes-Weinstein 5.07 monofilament.

Radiographs and computed tomography of the right foot revealed a Charcot homolateral tarsometatarsal joint dislocation, medial displacement of the navicular, inferior subluxation of the tarsometatarsal joints, as well as hypertrophic osseous growth and fragmentation at the first and second proximal metatarsal shafts and along the medial navicular. Noninvasive vascular testing showed no evidence of significant arterial disease. Laboratory testing was unremarkable except for elevation of serum glucose (146 mg/dL). Chest x-ray and electrocardiogram were within normal limits.

After discussion with the patient about all possible treatment options and perioperative considerations, the patient elected to have surgical correction of the CN foot deformity. He was medically optimized and cleared for surgery. He was given intravenous clindamycin preoperatively for infection prophylaxis. Under general endotracheal anesthesia and ipsilateral pneumatic thigh tourniquet, a curvilinear 8 cm medial incision from the first metatarsal to the medial malleolus was made. Dissection was carefully carried out, with care to maintain a full-thickness dorsal and medial flap. The naviculocuneiform and metatarsocuneiform joints were located. The medial cuneiform was noted to be subluxed medially with respect to the naviculocuneiform joint.

**Figure 1:**
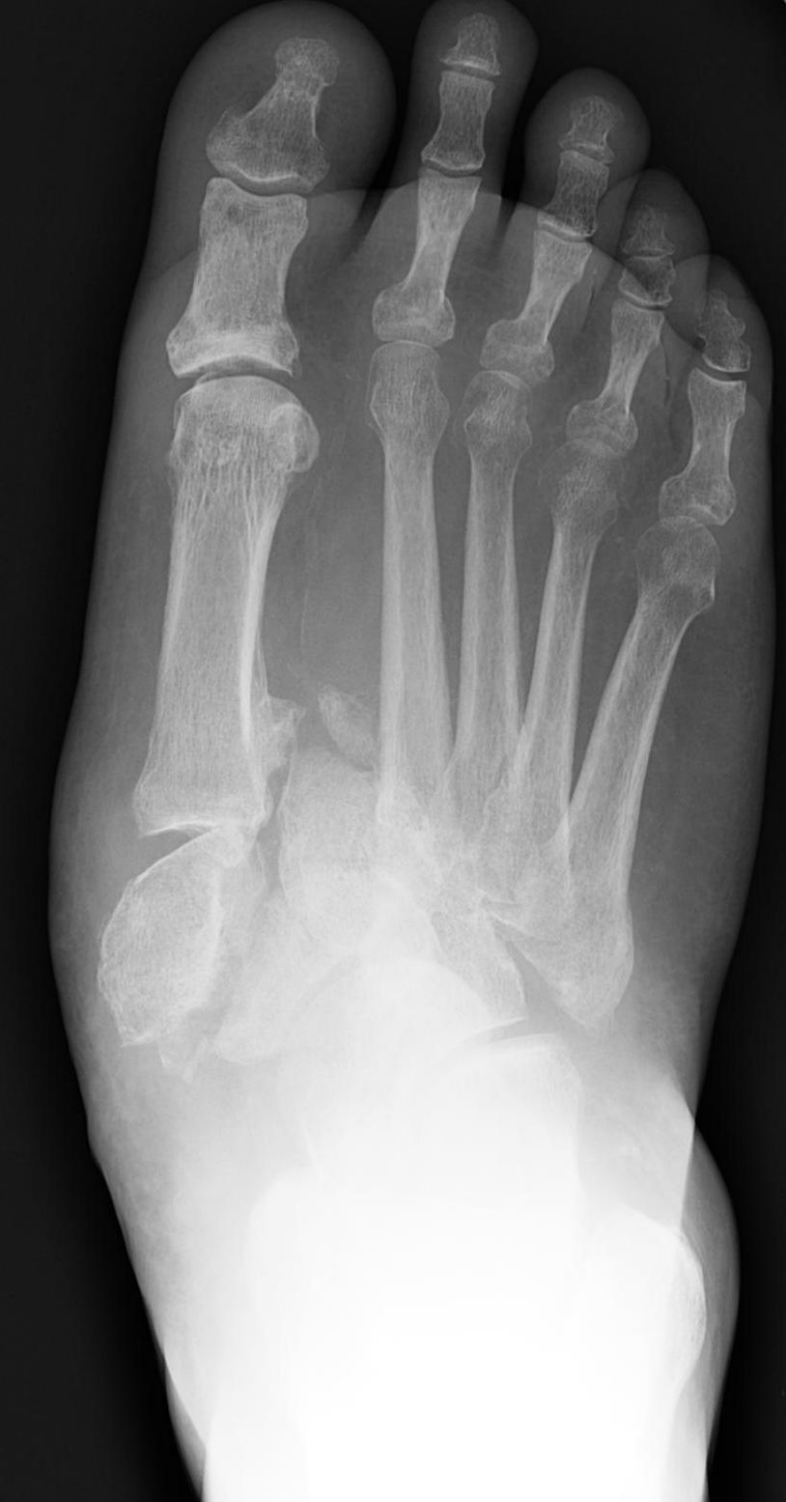
**Preoperative anteroposterior radiographic view showing the severe midfoot fracture-dislocation of the diabetic CN foot**.

**Figure 2:**
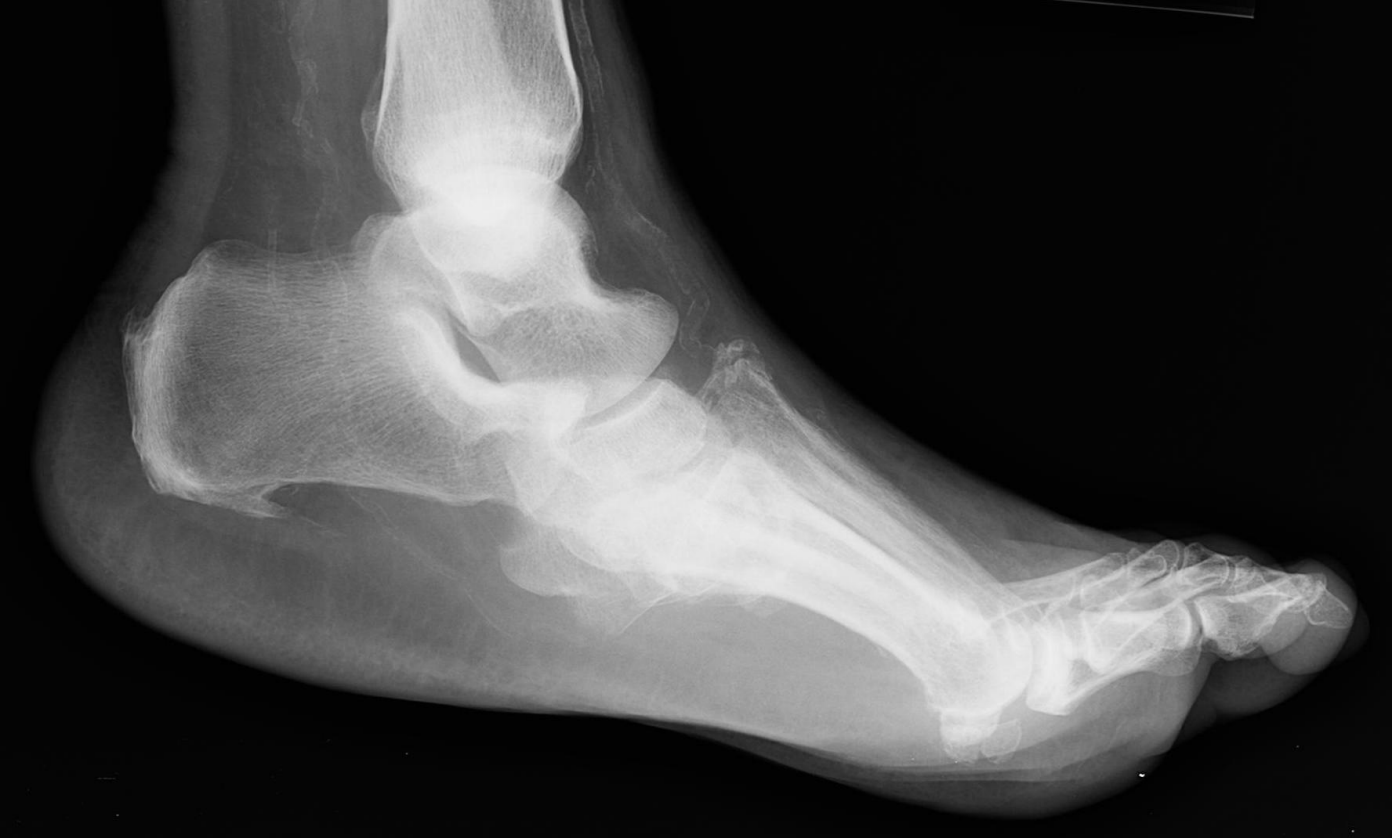
**Lateral radiographic view showing the severe midfoot fracture-dislocation of the diabetic CN foot**.

**Figure 3:**
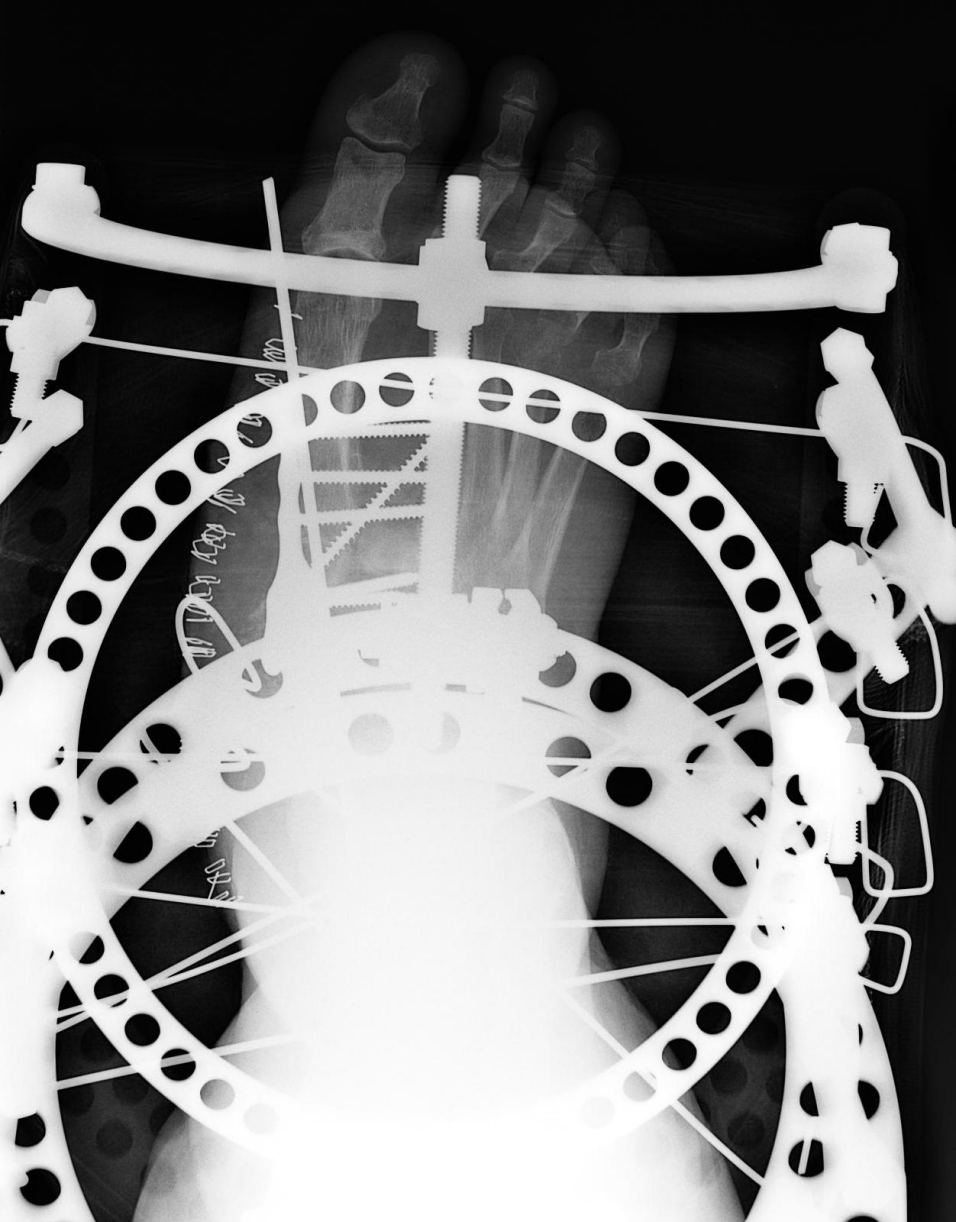
**Postoperative anteroposterior radiographic view showing the multiple midfoot arthrodesis sites with combined internal and external fixation methods**.

**Figure 4:**
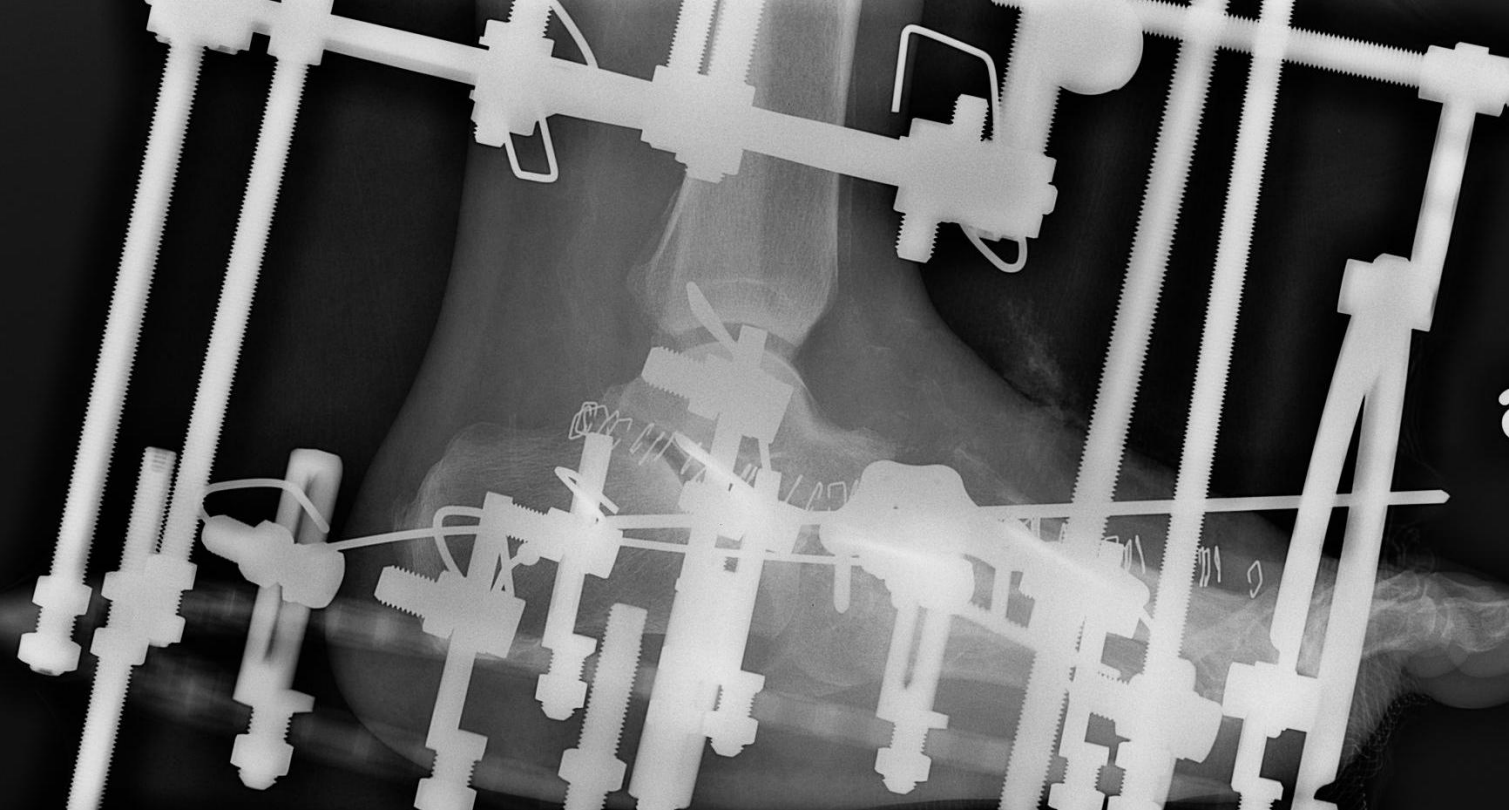
**Lateral radiographic views showing the multiple midfoot arthrodesis sites with combined internal and external fixation methods**.

**Figure 5:**
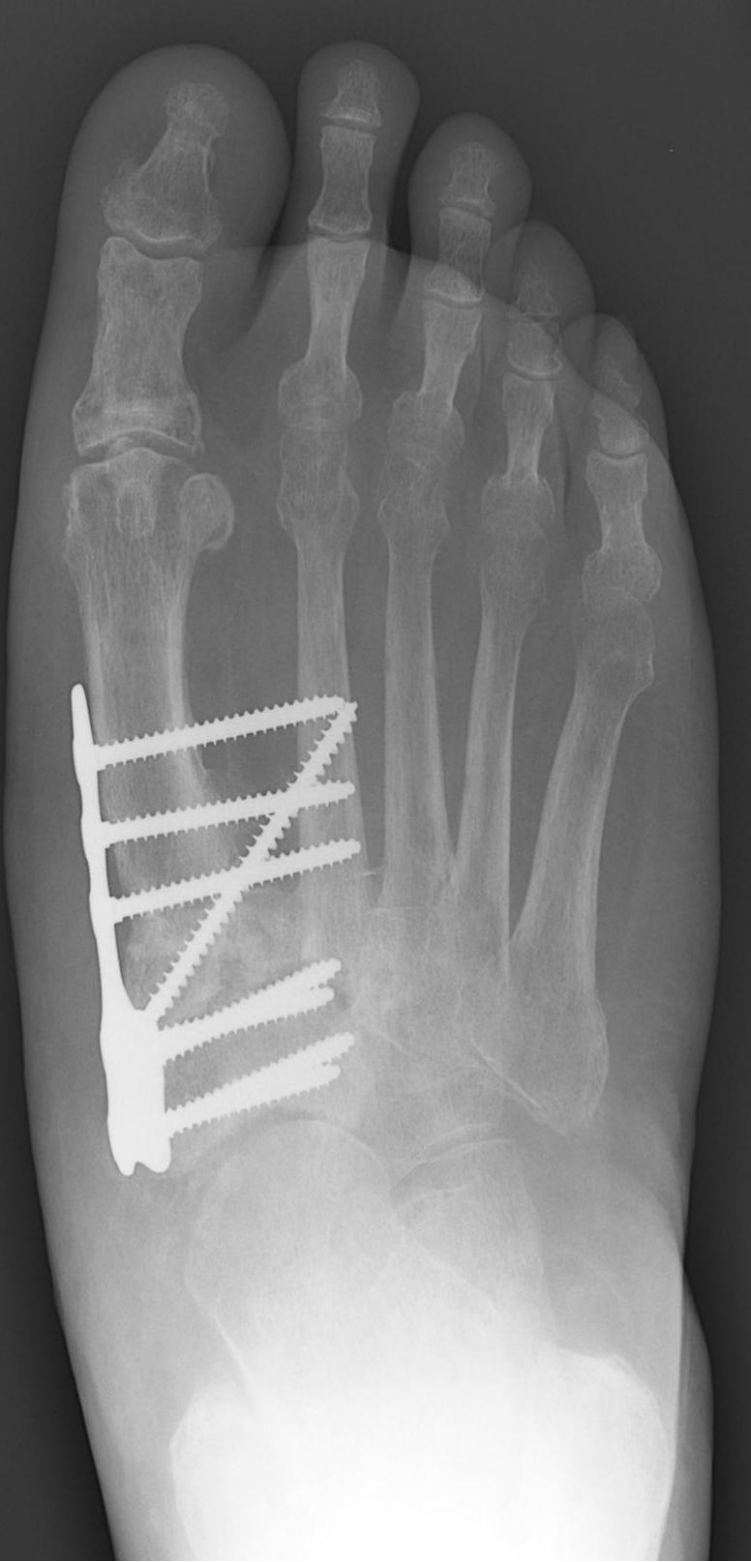
**Final one year follow-up anteroposterior radiographic view showing anatomic alignment and consolidation across the arthrodesis sites**.

**Figure 6:**
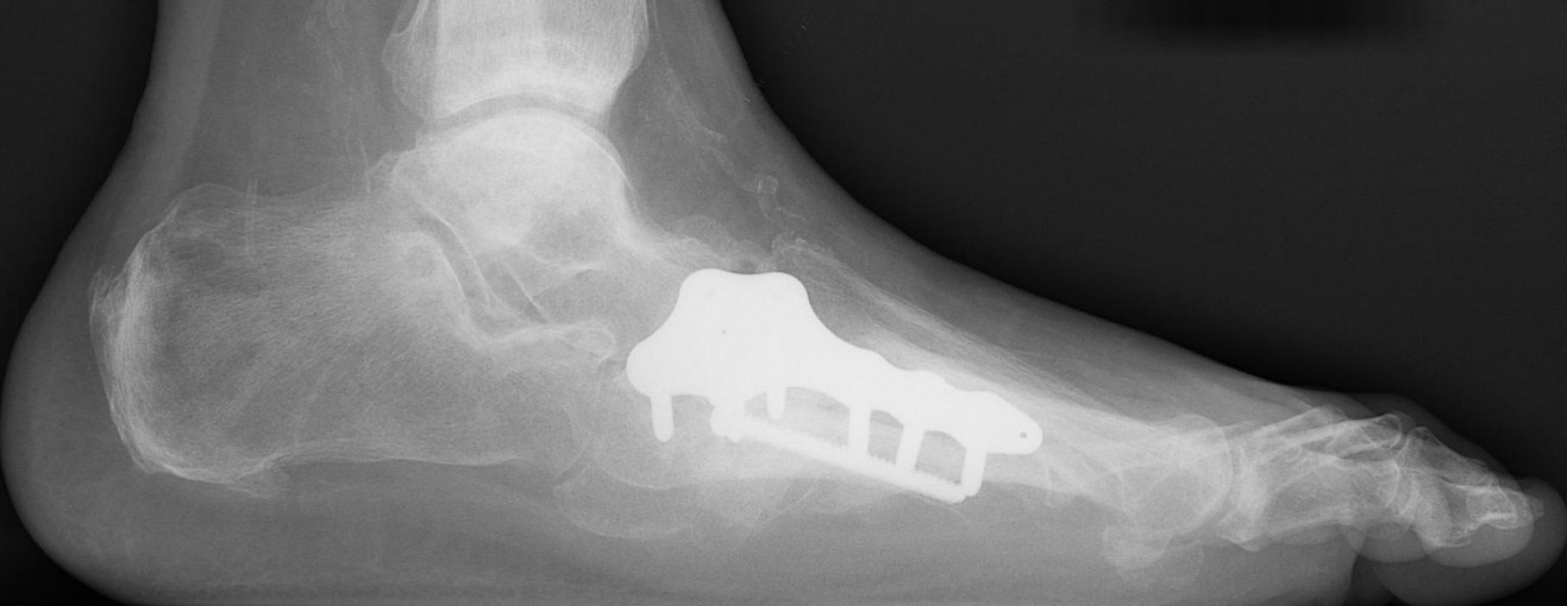
**Lateral radiographic view showing anatomic alignment and consolidation across the arthrodesis sites**.

The joints were identified, capsulotomies performed and their interosseous ligaments transected to allow for mobilization and deformity correction. After thorough removal of opposing articular surfaces, the joints were manipulated into a corrected position with simulated weight bearing and were temporarily fixated with 2.8 mm Steinmann pins. Intraoperative fluoroscopy was utilized to assess for adequate reduction of the deformity. Next, 5 cc of morselized allogenic bone graft was packed into the arthrodesis sites and a medial column locking plate was sized and contoured. Fully threaded cortical non-locking 3.5 mm screws were placed in the most proximal and distal holes of the plate so as to minimize stress risers at these locations. A fully threaded 4.0 mm cortical screw was inserted utilizing lag technique to restore the Lisfranc ligament. Fully threaded 3.5 mm cortical locking screws were placed in the remainder of the plate, with sufficient length so as to capture the intermediate cuneiforms and lesser metatarsal bases for additional construct stiffness. The incision was then closed in layers, taking care to cover the hardware with deep capsule and fascia, and the tourniquet was deflated. Next, after a sterile re-preparation of the ipsilateral limb, the prebuilt Ilizarov circular external fixator was positioned appropriately with respect to the right lower extremity. After appropriate positioning, frontal and oblique plane wires were inserted and secured to the external fixator for further stabilization and compression. Post-operative radiographs demonstrated maintenance of the lower extremity alignment (Figures 1, 2, 3, 4, 5, 6).

The patient was prophylaxed for deep venous thrombosis and kept on strict bedrest for three days postoperatively. On post-operative day four, he worked with physical therapy on transfers to chair while maintaining strict non-weight bearing status to the operative limb. He was cleared for discharge to a rehabilitation facility for strengthening, and was discharged home one week later.

The patient was seen at post-operative week one for a dressing change, and every two weeks thereafter for incision and external fixation care. Radiographs demonstrated bony bridging at post operative week six and the patient underwent an uncomplicated post-operative course. He was taken back to the operating room at post-operative week eight for removal of the external fixator and application of a non-weight bearing below the knee fiberglass cast. He remained non-weight bearing for eight weeks, and subsequently began weight bearing in a walking boot for six additional weeks. At post-external fixator removal week twelve, the patient was transitioned into a custom double-upright brace, and underwent incremental increases in activity level over the next six months. At one year after the initial surgery, the patient was ambulatory in diabetic extra depth shoes, without evidence of soft tissue or osseous breakdown.

## Discussion

Options for surgical management of patients with CN range from simple exostectomy with ulcer excision to major reconstruction with arthrodesis, internal and external fixation. The authors advocate a highly individualized treatment plan according to each patient's specific manifestations of the disease process. The authors support reconstructive surgery for unstable and progressive deformity in the setting of ulceration or pre-ulceration, with or without evidence of osteomyelitis. The staging of reconstruction after eradication of osteomyelitis, if present, is essential. Combined internal and external fixation is often preferred for complex reconstruction in the severely deformed insensate foot. Additionally, stabilization of adjacent joints with external fixation has been described, and may also be employed. Furthermore, if plastic coverage is necessary to close plantar, medial or lateral wounds after ulcer excision and reconstruction, use of adjunctive external fixation aids in offloading the flap or skin graft.

Isolated exostectomy of plantar bony prominences is common, and has been reported to be quite successful if performed after the deformity has consolidated [14,15]. Reactivation of CN pathology in the ipsilateral foot may occur in up to 15% of patients [16]. Recurrent instability or continued overloading of the affected area may result in recurrence of the ulcer and warrant more substantive intervention.

Reconstructive surgery of the Charcot foot typically entails stabilization and/or arthrodesis of multiple collapsed joints. Plantar exostectomy, plastic coverage and/or posterior muscle group lengthening are often performed concomitantly [17]. Medial and lateral column arthrodesis may be performed with large medial and lateral column screws [13,18,19], bolts, or plates [20]. Currently, no side-by-side comparison of fixation methods for Charcot foot and ankle reconstruction exists in the literature. Complications after CN foot reconstruction are frequent [21] and include hardware failure, deep and superficial infection, wound dehiscence, pseudoarthrosis, instability, and amputation [8].

External fixation has been described in the literature as a primary or adjunctive procedure for Charcot foot and ankle reconstruction [13,22-25]. The technique allows stress shielding of the affected arthrodesis sites and also augments the bending stiffness and torsional resistance of the overall construct. The presence of the external fixator may also act as an additional deterrent for inappropriate weight bearing on the operative limb. Potential complications associated with the use of external fixation include and are not limited to: pin or wire tract infections, hardware failure requiring premature discontinuation of the external fixator, stress fractures, osteomyelitis and difficulty psychologically acclimating to the device. Pin and wire complications are widely known as the most frequent complication in application of external fixation devices in any patient population. In a retrospective study evaluating circular ring external fixation, Wukich et al related a seven-fold increase in wire complications in diabetic patients versus non-diabetics [26]. Proper early identification, mitigation, and treatment of these complications are essential to success of the reconstruction.

Lower extremity amputation is known to affect a significant increase in cardiovascular output, which is highly significant in the patient population affected by CN. The majority of these patients have multiple end-organ sequelae of uncontrolled diabetes mellitus, including severe peripheral vascular compromise and often silent coronary artery disease. Prudent multi-disciplinary evaluation on a case-by-case basis of the risk-benefit ratio of lower extremity reconstruction and salvage versus amputation is fundamental and in the best interest of the patient. Candid discussions with the patient and family members about the critical protracted non-weight bearing period, potential complications, and frequent visits following reconstruction are essential.

## Conclusion

Successful surgical treatment of the CN foot is predicated on reducing deformity, stabilizing adjacent joints, and removing osseous prominences. The authors describe their preferred surgical management of unstable, progressive and non-infected CN foot and ankle deformities with a combination of internal and external fixation, which provides both stability and compression across the arthrodesis sites. Deliberate restraint and frequent follow-up are crucial during resumption of protected weight bearing for these patients. Ultimately, after the predictably protracted post-operative course, most patients are able to return to diabetic shoe gear or braces with long-term activity modification.

## Consent

Written informed consent was obtained from the patient for publication of this case report and any accompanying images. A copy of the written consent is available for review by the Editor-in-Chief of this journal.

## Competing interests

The authors declare that they have no competing interests.

## Authors' contributions

CMC performed part of the literature review and contributed in drafting of the manuscript. CLR performed part of the literature review and assisted in the case report presentation. TZ conceived the idea of the present study, performed part of the literature review and contributed in the manuscript editing. All authors have read and approved the final manuscript.
